# Relationship between emotional intelligence and quality of healthcare among nurses

**DOI:** 10.3389/fpsyg.2024.1423235

**Published:** 2024-10-23

**Authors:** Islam Oweidat, Mahmoud Alzoubi, Ghada Abu Shosha, Wafa’a Ta’an, Anas Khalifeh, Majdi M. Alzoubi, Khalid Al-Mugheed, Amany Anwar Saeed Alabdullah, Sally Mohammed Farghaly Abdelaliem

**Affiliations:** ^1^Community and Mental Health Nursing Department, Faculty of Nursing, Zarqa University, Zarqa, Jordan; ^2^Department of Clinical Nursing, Faculty of Nursing, Zarqa University, Zarqa, Jordan; ^3^Department of Community and Mental Health Nursing, Faculty of Nursing, Jordan University of Science and Technology, Irbid, Jordan; ^4^Faculty of Nursing, Al-Zaytoonah University of Jordan, Amman, Jordan; ^5^College of Nursing, Riyadh Elm University, Riyadh, Saudi Arabia; ^6^Department of Maternity and Pediatric Nursing, College of Nursing, Princess Nourah Bint Abdulrahman University, Riyadh, Saudi Arabia; ^7^Department of Nursing Management and Education, College of Nursing, Princess Nourah Bint Abdulrahman University, Riyadh, Saudi Arabia

**Keywords:** emotional intelligence, health, nurse, quality, quality of healthcare, nurses

## Abstract

**Introduction:**

Healthcare organizations worldwide face intense competition for survival in an ever-changing environment.

**Objectives:**

This study aims to examine the relationship between emotional intelligence (EI) and quality of healthcare (QHC) among Jordanian nurses working in governmental hospitals.

**Method:**

This study was conducted using a descriptive correlational design and included a sample of 172 nurses. Participants from five governmental hospitals in Jordan completed online self-administered questionnaires, including the Nurse-Assessed Quality of Nursing Care Scale and the Genos Emotional Intelligence Concise Scale, along with sociodemographic data.

**Results:**

The participating nurses demonstrated a high level of EI (M = 3.809, SD ± 0.484) and a very high level of QHC (M = 4.260, SD ± 0.372). A statistically significant correlation was found between the total quality of healthcare variables and the total EI variable (*r* = 0.739, *p* < 0.01). Additionally, statistically significant correlations were observed between the QHC and EI, as well as their respective dimensions (*r* = 0.357–0.739). EI was found to be a significant predictor of the QHC (*F* = 34.872, *p* ≤ 0.001), with a positive correlation between the two variables (*r* = 0.733). EI accounted for 59.8% of the variation in the QHC.

**Conclusion:**

EI is a key predictor of QHC. It plays an essential role in recruiting, staffing, promoting, and nurturing employees, making it a crucial criterion for achieving excellence in healthcare organizations.

## Introduction

Healthcare organizations, similar to other businesses operating worldwide, face intense survival competition not only in commercial and trading fields but also in retaining their competent employees amidst this competition (Sullivan and Decker, 1998). Employees at all levels are the cornerstone of every organization, especially in healthcare settings. Creating a positive and healthy work environment where staff can develop, thrive, and succeed is essential and should be a key element in the development plan of every healthcare organization (Al-Oweidat et al., 2023).

The unfamiliarity with the concept of emotional intelligence (EI) leads to its irregular and unsystematic use, which diminishes the expected benefits in the healthcare context (Fariselli et al., 2008). Moreover, nurses’ EI and stability play crucial roles in improving the quality of healthcare (QHC). Unfortunately, in some healthcare specialties, this quality tends to decrease over time. EI and self-compassion are significant elements, especially when looking after mentally ill patients (Codier et al., 2013; Kousar et al., 2017).

Al-Hamdan et al. conducted a study in Jordan to examine the relationship between EI and nurse-to-nurse collaboration among Jordanian registered nurses. Nurses’ EI was significantly and positively correlated with the nurse-to-nurse collaboration subscales. The study demonstrated that nurse-to-nurse collaborations are linked to numerous benefits for registered nurses, including improved job quality, patient care, satisfaction, better nurse retention, and enhanced healthcare productivity and efficiency. This finding suggests that improving nurse-to-nurse collaboration could yield significant advantages not just for staff nurses and organizations but also for patients and the overall healthcare system (Al-Hamdan et al., 2021).

In Iran, a descriptive correlational study was performed to investigate the relationship between EI and quality of nursing care from both nurses’ and patients’ perspectives (Khademi et al., 2021). The findings of the study reveal EI positively affects the quality of nursing care and its dimensions. Therefore, it is highly recommended that nursing policymakers consider educational programs to strengthen nurses’ EI and enhance the quality of nursing care.

The current study will expand our knowledge regarding the impact and influence of EI on QHC among healthcare providers. Similarly, the findings will be helpful for hospital management, policymakers, and government officials in developing strategies to provide better healthcare services for all patients. The results will also help nurse managers explore strategies that can enhance nurses’ EI and improve their QHC levels.

This study was conducted to examine the relationship between EI and QHC among Jordanian nurses in governmental hospitals, addressing the following hypotheses: What are the levels of EI and QHC among Jordanian nurses in governmental hospitals? Is there a relationship between the EI and QHC among Jordanian nurses in governmental hospitals? What are the predictors of EI and QHC levels among Jordanian nurses working in governmental hospitals?

## Methodology

### Design

A descriptive, cross-sectional correlational design using a self-reported questionnaire was used to achieve the study objectives.

### Participants and context

The target population for this study included all Jordanian nurses employed in Jordanian governmental hospitals, namely Al Basheer Hospital, Zarqa Governmental Hospital, Al Karak Governmental Hospital, Mafraq Governmental Hospital, and Princess Basma Hospital. The accessible population included nurses working in hospitals with an Internet connection who could use the Internet on either smart devices or computers and were interested in filling out the study questionnaires. A convenience sampling method was used to recruit the study participants. This involved the author meeting with the unit managers in each hospital. Then, we sent a Google Form link to the unit’s group after discussing the inclusion and exclusion criteria with the manager. The inclusion criteria were as follows: being a nurse (registered nurse or clinical nurse specialist), holding at least a bachelor’s degree, being willing to voluntarily participate in the study, and working in direct patient care in any hospital unit. Participants were unable to submit the survey unless all fields were completed. They could only submit the survey once.

The author calculated the sample size according to Thompson’s equation, determining that a minimum of 119 participants were required. Additionally, the target number of participants was increased to 150 to avoid the possibility of missing data. With the application of the e-survey method, it was not possible to estimate the response rate. The author planned to invite nurses to participate in the study by keeping the survey link active for 2 months. The response rate was regularly monitored, and once the minimum required sample size was reached, the remaining invitations were closed by deactivating the survey link. If necessary, a time extension was granted. At the final check, a total of 172 participants had completed the survey. Subsequently, the researcher deactivated the survey link and began the statistical data analysis.

### Instruments

The sociodemographic characteristics of the participants included age, gender, educational level, marital status, years of experience, and work unit (see Table 1 for details). The Nurse-Assessed Quality of Nursing Care Scale (QNCS) comprises 48 items or statements describing the nursing services provided to patients. Participants were encouraged to carefully read each statement and select their response using a scale from 1 to 5, where 1 = Fully Disagree, 2 = Disagree, 3 = Unsure, 4 = Agree, and 5 = Fully Agree. The scale emphasized that there were no right or wrong answers, allowing participants to select their choices freely. No items required reverse coding before calculation, as all items were oriented in the same direction. In this study, the Cronbach’s *α* value for the scale was 0.81, indicating good reliability.

**Table 1:** Sociodemographic characteristics of the participants (*n* = 172).

Variable		Number	Percentage (%)
Age	20–29 years	65	37.79
	30–39 years	71	41.28
	40–55 years	36	20.93
Gender	Men	100	58.14
	Women	72	41.86
Educational level	Bachelor	130	75.58
	Postgraduate degree	42	24.42
Marital status	Married	110	63.95
	Single	50	29.07
	Divorced/Widowed	12	6.97
Experience	1–5 years	30	17.44
	6–10 years	43	25.00
	11–15 years	46	26.74
	16–20 years	34	19.77
	21 years or more	19	11.05
Work unit	Acute care unit	138	80.2
	Critical care unit	34	19.8

Genos emotional intelligence survey comprises a series of 31 statements that were also used to measure the EI variable. The participants were asked to circle the number that best corresponds to how they think, act, and feel at work. The response options ranged from 1 to 5, where 1 = Almost Never, 2 = Seldom, 3 = Sometimes, 4 = Usually, and 5 = Almost Always. Participants were instructed to focus on their typical behaviors, rather than specific situations, when selecting their responses. In this study, the survey demonstrated good reliability, with a Cronbach’s *α* value of 0.85.

### Data collection

The study participants remained anonymous, and no names or identifying information were recorded. The participants were informed about the study’s purpose, procedure, possible risks or discomforts, and their rights. Participation was voluntary, and the participants had the right to withdraw at any point or time without excuses, explanations, or penalties. Informed consent was obtained implicitly when participants responded to the online questionnaire. All participants were informed that their responses and information would be kept confidential and private by the researcher. Data were collected after receiving ethical committee approval from the Institutional Review Board (IRB) of the Faculty of Nursing at Zarqa University, as well as from the Jordanian Ministry of Health (MOH). The questionnaires were distributed online as planned. The data collection process was carried out for two months. The online surveys were sent via Google Forms to the online nursing groups, and they contained a participation invitation letter of acceptance. All survey data fields were mandatory, meaning participants could not submit the survey if any part was incomplete. The information letter informed the study participants that they could only complete the survey at their convenience once.

### Data analysis

Data were analyzed using the Statistical Package for the Social Sciences (SPSS), version 26. The statistical analyses included calculating frequency and percentages to describe categorical variables (e.g., gender and education level). For continuous variables (e.g., age and EI scores), means and standard deviations were calculated to provide a summary of the central tendency and dispersion. The relationship between EI and QHC was examined using Pearson’s r coefficient to assess the strength and direction of the linear relationship between these two continuous variables. A significance level of *p* < 0.05 was set for all analyses.

## Results

### Participants characteristics

A total of 172 registered nurses participated in this study. As shown in Table 1, the most common age group of participants was between 30 and 39 years (*n* = 71, 41.28%). Most of the participants were men (*n* = 100, 58.14%), married (*n* = 110, 63.95%), and held a bachelor’s degree in nursing (*n* = 130, 75.58%).

In terms of work department, the majority of the study participants (*n* = 138, 80.2%) worked in acute care units, including emergency, medical, and surgical units, whereas only 34 participants (19.8%) worked in critical care units, including adult, pediatric, and neurological ICUs. The most common years of experience range was between 11 and 15 years (*n* = 46, 26.74%), while only 19 participants (11.05%) had 21 years of experience or more.

The results, as shown in Table 2, revealed that the participants reported a high level of EI (mean = 3.81, SD = 0.48) and a very high level of quality of nursing care (mean = 4.260, SD = 0.37). The minimum, maximum, and mean values of the dimensions of EI and QNC, as shown in Table 2, demonstrate relatively close values for all dimensions of the two variables.

**Table 2:** Descriptive statistics of the EI and QNC total scores and domains (*n* = 172).

Variable	Min.	Max.	Mean	St. D	Level
Total quality of nursing care (QNC)	3.35	5.00	4.26	0.37	Very high
Task-oriented activities	3.07	5.00	4.23	0.37	Very high
Staff characteristic	3.13	5.00	4.24	0.44	very high
Physical environment	3.00	5.00	4.29	0.46	Very high
Human-oriented activities	3.43	5.00	4.33	0.40	Very high
Precondition	2.14	5.00	4.21	0.48	Very high
Patient outcomes	2.67	5.00	4.31	0.46	Very high
Total emotional intelligence (EI)	2.90	4.71	3.81	0.48	High
Emotional self-awareness (ESA)	2.50	5.00	3.89	0.75	High
Emotional expression (EE)	2.60	4.40	3.50	0.41	High
Emotional awareness of others (EAO)	2.50	5.00	3.95	0.67	High
Emotional reasoning (ER)	2.20	5.00	4.00	0.68	High
Emotional Self-management (ESM)	2.60	5.00	3.90	0.58	High
Emotional management of others (EMO)	2.50	5.00	3.90	0.65	High
Emotional self-control (ESC)	2.25	4.75	3.53	0.49	High

The results of the Pearson correlation coefficient test in Table 3 revealed a statistically significant correlation (0.01) between the total QNC scores and the total EI scores, which amounted to 0.74. It is also clear from the previous table that there is a statistically significant correlation at the level of significance (0.01) between the variable QNC in general and each of EI’s dimensions, as well as with the EI variable in general and each of QNC dimensions. The Pearson correlation coefficients (*r*) ranged between 0.36 and 0.74, with all coefficients statistically significant at the 0.01 level.

**Table 3:** Pearson correlation coefficients between the study variables.

	Total EI	ESA	EE	EAO	ER	ESM	EMO	ESC
Total QNC	0.74^**^	0.60^**^	0.45^**^	0.65^**^	0.69^**^	0.62^**^	0.62^**^	0.43^**^
Task-oriented activities	0.67^**^	0.51^**^	0.42^**^	0.60^**^	0.63^**^	0.57^**^	0.55^**^	0.40^**^
Staff characteristic	0.67^**^	0.58^**^	0.36^**^	0.57^**^	0.65^**^	0.55^**^	0.59^**^	0.37^**^
Physical environment	0.66^**^	0.53^**^	0.42^**^	0.58^**^	0.64^**^	0.54^**^	0.58^**^	0.37^**^
Human-oriented activities	0.61^**^	0.52^**^	0.36^**^	0.55^**^	0.57^**^	0.50^**^	0.50^**^	0.36^**^
Precondition	0.64^**^	0.51^**^	0.41^**^	0.55^**^	0.58^**^	0.53^**^	0.57^**^	0.36^**^
Patient outcomes	0.62^**^	0.54^**^	0.40^**^	0.55^**^	0.53^**^	0.54^**^	0.47^**^	0.36^**^

**Correlation is significant at the 0.01 level (2-tailed).

A multiple regression analysis was conducted, considering EI and demographic data as independent variables to examine their impact on the quality of nursing care, which is a dependent variable. The overall prediction model, presented in Table 4, demonstrated good performance, with an R2 of 0.66, indicating that approximately 66% of the variation in nursing care quality can be explained by the independent variables included in the model. The adjusted R2 value of 0.64 further confirms the model’s reliability in predicting nursing care quality.

**Table 4:** The prediction model for quality of nursing care.

Variables	*B*	Std. error	β	*t*
(Constant)	2.225	0.156		14.279**
Gender	0.064	0.035	0.085	1.810
Age	0.053	0.044	0.113	1.201
Marital status	0.007	0.026	0.013	0.258
Educational level	0.065	0.030	0.102	2.177*
Work Unit	−0.027	0.008	−0.159	−3.379**
Experience	0.049	0.030	0.165	1.627
Total EI Score	0.452	0.044	0.588	10.300**

Model summary				
	R^a^	R^2^	R^2^_adj_	Std. error of the estimate
	0.81	0.66	0.64	0.22

Model ANOVA				
	Sum of squares	df	Mean square	*F*
	15.54	7	2.22	45.00**

Dependent variable: quality of nursing care. *N* = 172, * *p*-value < 0.05, ** *p*-value < 0.01.

The Model ANOVA results showed that the model is highly significant (*F* = 45.00, *p* < 0.01), providing evidence for the overall predictive power of the model.

In conclusion, this multiple regression analysis provides valuable insights into the factors influencing the quality of nursing care. The findings highlight the importance of EI, education, and work units in shaping the overall quality of care provided by nurses. Understanding these associations can assist in developing targeted interventions and strategies to enhance nursing care quality and ultimately improve patient outcomes.

The results reveal interesting insights. Educational levels had a significant positive impact on nursing care quality (*β* = 0.102, *t* = 2.177, *p* < 0.05). This suggests that higher levels of education among nurses are associated with improved nursing care quality. Interestingly, the work unit was found to have a negative and substantial influence on nursing care quality (*β* = −0.159, *t* = −3.379, *p* < 0.01). This implies that nurses working in certain units may encounter challenges that negatively affect the quality of care they provide. Moreover, the “Total EI Score,” representing the total score on an EI assessment, displayed the most substantial positive relationship with nursing care quality (*β* = 0.588, *t* = 10.300, *p* < 0.01). This finding indicates that nurses with higher EI scores are more likely to deliver better-quality nursing care.

Among the independent variables, gender had a positive but weak association with the quality of nursing care (β = 0.09, *t* = 1.81, *p* > 0.05). Age had a slightly stronger positive relationship (β = 0.113, *t* = 1.201, *p* > 0.05). In contrast, marital status showed a negligible effect (β = 0.013, *t* = 0.258, *p* > 0.05). The variable “experience” was found to have a moderately positive impact on nursing care quality (β = 0.165, *t* = 1.627, *p* > 0.05). These results suggest that as nurses gain experience, the quality of care they provide tends to improve. However, gender, age, marital status, and experience were not found to be statistically significant predictors of QNC.

## Discussion

### The main findings of this study

The results of this study showed that the participants generally exhibited high levels of EI, as reflected in their mean scores across all seven dimensions of EI. The highest subscale score was for emotional reasoning (ER), which refers to the ability to use information from one’s own feelings and the feelings of others when making decisions.

This involves considering both emotional and factual information, then effectively communicating the decision-making process to others” (Palmer et al., 2009). The second-highest score was for emotional awareness of others (EAO), which refers to the ability to perceive, acknowledge, and understand how others feel. The findings are similar but slightly lower compared to the results of a study performed in Jordan, which revealed that the Jordanian nurses in a magnet hospital reported an overall mean EI score of 5.60 out of 6.0. It and proved the predictive ability of the EI variable in the practice field among Jordanian nurses (Al-Ruzzieh and Ayaad, 2021).

The results of this study showed that participants achieved a very high level of QNC overall, as well as across the six specific factors of QNC. The highest mean score was for factor four, *human-oriented activities*, closely followed by factor six, *patient outcomes*, with only minimal differences in mean scores between the two factors. In a related dyadic study, findings indicated that nurses’ perceptions of QNC were higher than those of patients, and certain factors related to both nurses and patients negatively influenced the quality of care (Mert et al., 2021).

Examining the relationship between EI and QHC among Jordanian nurses in this study revealed a significant positive correlation between the variables of EI and QHC, along with their respective dimensions. Studies support this relationship, indicating that EI plays a crucial role in influencing healthcare outcomes. Many researchers assert that EI is important and has significant effects on our lives, particularly in leadership roles (Momm et al., 2015). EI is usually analyzed in the context of its impact on interpersonal relationships, such as the relationship between leaders and employees. Leadership is usually interpreted as a process of influencing others, motivating employees, setting goals, and driving high performance (Krén and Séllei, 2021).

According to Rode et al., emotions play an essential part in leadership, with leaders managing the moods and emotions of their community as key figures in their communities (Rode et al., 2017). Karimi et al. found that higher levels of EI significantly were associated with better results in patient care quality and improved health professional wellbeing (Karimi et al., 2021). The relationship between EI and quality of care was tested, revealing that total EI significantly correlated with the quality-of-care variables, including *Clostridioides difficile* infections, methicillin-resistant *Staphylococcus aureus* (MRSA) infections, and patient falls with injury (Adams and Iseler, 2014). EI also predicts employees’ wellbeing, psychological empowerment, and quality of care. Moreover, this study indicated that employees with higher EI are more likely to deliver higher-quality patient care (Mderis et al., 2024). Effective and strong leadership is fundamental to organizational competency and significantly influences care quality (Mansel and Einion, 2019; Oweidat et al., 2024).

The Pearson correlation coefficients between the study variables showed that all correlation coefficients were statistically significant. Among the dimensions of the independent variable EI, the current study findings found that the *Emotional Awareness of Others* (EAO) dimension was the strongest predictor, explaining the majority of the variance in the QHC variable. The Emotional Reasoning (ER) dimension accounted for the remaining variance after controlling for the effect of EAO. Nurses with high levels of EAO demonstrate a strong ability to understand what makes people feel optimistic and valued at work. They are also skilled at recognizing how others respond, building rapport, and identifying factors that motivate people with fewer challenges. This relates to their ability to empower, encourage, and motivate others, which helps influence their interactions and relationships. These skills are directly tied to improving the QHC by enhancing key components such as effective communication, satisfaction, loyalty, empathy, compassion, and motivation among healthcare providers (Navarro-Bravo et al., 2019; Alzoubi et al., 2024a). Nurses who score high in the ER dimension have a strong ability to capture others’ attention when communicating decisions and consistently consider others’ feelings and reactions during the decision-making process at work.

The impact of EI enhances and strengthens investments in shared governance, staff involvement, and patient-centered care.

Nurses play a crucial role in improving workplaces by securing stakeholder commitment to their decisions, thereby contributing to action and strategic planning within their organizations (Sanchez-Gomez et al., 2021). Effective communication of their decisions and feedback, along with its benefits, is essential for gaining this commitment. Other studies demonstrate that nurses with strong social skills and relationship management tend to exhibit higher EI and report greater job satisfaction (Adams and Iseler, 2014). Similarly, colleagues, patients, families, and others who interact with nurses who possess high EI often find their experiences more satisfying, leading to more positive healthcare outcomes for both nurses and patients. Nurses who actively develop their EI intentionally create an environment where patients, families, and colleagues feel genuinely cared for and supported (White and Grason, 2019; Alzoubi et al., 2024b; Ishii and Horikawa, 2019).

### Implications of these results in the field of research study

The findings of this study highlight the significant role of EI in improving the QHC and outcomes. The positive impact of EI extends to enhanced employee engagement and involvement, contributing to better patient care and organizational efficiency. Furthermore, EI significantly reduced undesirable behaviors, such as conflict, within healthcare settings.

### These results highlights the need for healthcare organizations to prioritize EI in several key areas

#### Recruitment and staffing

Incorporating EI assessment in the recruitment and staffing processes is essential. Healthcare organizations should prioritize candidates with high EI to foster a positive work environment and improve patient outcomes. This strategy will also help build a workforce capable of handling healthcare’s emotional and interpersonal demands.

#### Training and development

The development of EI-focused training programs and continuous education is crucial. Regular, pre-scheduled training sessions and workshops on EI will help healthcare employees strengthen their emotional and interpersonal skills. This, in turn, will enhance their capacity to manage stress, communicate effectively, and collaborate within teams, all of which are vital to improving the QHC.

#### Skill competencies

Developing EI competencies through consistent education and practice is critical for maintaining high-quality performance in healthcare. Institutions should implement ongoing training to not only build knowledge and skills but also improve healthcare staff’s attitudes and behaviors. This approach will contribute to long-term improvements in both employee wellbeing and organizational outcomes.

### Future lines of research

While this study highlights the significant influence of EI on QHC, future research should explore the long-term effects of EI training on healthcare outcomes and employee wellbeing. Longitudinal studies can be conducted to track changes in healthcare quality, patient satisfaction, and staff performance following the implementation of EI-focused programs over an extended period. Additionally, understanding how emotionally intelligent leaders influence team dynamics, employee satisfaction, and patient care may provide valuable insights indeveloping more effective healthcare leadership models.

## Limitations

Although the study has several strengths, it also has some limitations. First, the cross-sectional study design limits the ability to establish cause-and-effect relationships. Second, the study relied on online recruitment using a convenient sampling method, making it impossible to estimate the response rate accurately. Finally, the inclusion of only nurses from governmental hospitals may limit the generalizability of the findings to other healthcare settings.

## Conclusion

EI is a valuable skill that can help nurses deliver high-quality nursing care. Some of the results related to the dimensions of EI showed promising outcomes and demonstrated the predictability of the QHC among Jordanian nurses. As key members of healthcare teams, nurses are integral to a wide range of communication channels, coordinating roles and responsibilities. To succeed in this role, nurses must be able to accurately perceive, clearly understand, and effectively manage their own emotions, as well as the emotions of patients and their families, within the healthcare context.

## Data Availability

The raw data supporting the conclusions of this article will be made available by the authors, without undue reservation.
